# Diels–Alder reactions in confined spaces: the influence of catalyst structure and the nature of active sites for the retro-Diels–Alder reaction

**DOI:** 10.3762/bjoc.12.208

**Published:** 2016-10-13

**Authors:** Ángel Cantín, M Victoria Gomez, Antonio de la Hoz

**Affiliations:** 1 Instituto de Tecnología Química (UPV-CSIC), Universidad Politécnica de Valencia, Avda. Los Naranjos s/n, 46022 Valencia, Spain; 2 Área Química Orgánica, Facultad de Químicas, Universidad de Castilla-La Mancha, and Instituto Regional de Investigación Científica Aplicada (IRICA), Avda. Camilo José Cela s/n, E-13071-Ciudad Real, Spain

**Keywords:** catalysis, Diels–Alder, retro-Diels–Alder, zeolites

## Abstract

Diels–Alder cycloaddition between cyclopentadiene and *p*-benzoquinone has been studied in the confined space of a pure silica zeolite Beta and the impact on reaction rate due to the concentration effect within the pore and diffusion limitations are discussed. Introduction of Lewis or Brønsted acid sites on the walls of the zeolite strongly increases the reaction rate. However, contrary to what occurs with mesoporous molecular sieves (MCM-41), Beta zeolite does not catalyse the retro-Diels–Alder reaction, resulting in a highly selective catalyst for the cycloaddition reaction.

## Introduction

The Diels–Alder reaction (DAR) is one of the most useful reactions in organic synthesis. In order to improve the yield and to avoid the reversibility of the reaction, homogeneous Lewis acids [1–4], solid acids [5–6] as catalyst, high pressures [6–8] and/or water as a solvent [9–10] have been reported. In particular, and among the most interesting environmental-friendly reactions, the cycloaddition reaction occurs with high selectivity and atom economy. Moreover, Diels–Alder cycloadditions in combination with heterogeneous catalysts (i.e. doped-microporous materials) represent an interesting approach for the conversion of biomass feedstock into stable chemicals such as furfural derivatives, platform molecules which can be converted into a variety of liquid hydrocarbon fuels and fuel additives [11–12]. Catalysis is considered as one of the foundational pillars of green chemistry. Catalysis often reduces the energy requirements, permits the use of renewable feedstocks and less toxic reagents. Moreover, in most cases yields are improved and selectivity is enhanced or modified [13]. In this regard, heterogeneous catalysis in general and zeolites in particular are remarkably efficient since they permit the replacement of toxic mineral acids and oxidants by easily recyclable catalysts [14].

One approach to improve yields and selectivity is the special confinement of the reactants and the presence of catalytic active sites, [15–16] by use of microporous materials doped with metals. While pore dimensions and topology of the microporous materials can affect the selectivity of the reaction, their activity can be strongly limited by a slow diffusion of reactants and products, unless microporous molecular sieves with the appropriated pore dimensions are used as catalyst. Thus, microporous molecular sieves with optimized pore diameters and topologies can be of interest to catalyze DAR [17–26] in where different stereoisomers could be obtained. Lewis-acid centers contained within the framework of zeolite beta (Zr-β, Sn-β) are useful catalysts in the Diels–Alder reaction for the production of bio-based terephthalic acid precursors, one of the monomers for the synthesis of polyethylene terephthalate that is used for the large-scale manufacture of plastic bottles among others. The authors do not find transport limitations within the zeolite framework to the rate of the reaction [27]. Interestingly, when Brønsted acid containing zeolites (Al-β) are used as catalyst, there is a decrease in the Diels–Alder reaction selectivity [28].

The DAR of cyclopentadiene with *p*-benzoquinone is a well-known example of cycloadditions, and some results can be found on the control of the selectivity to the different isomers. In homogeneous phase, equimolar amounts of diene and dienophile afford two isomers, the *endo* as the major and the *exo* as the minor product. The addition of a second equivalent of cyclopentadiene affords mainly the *endo-anti-endo* product as major isomer, and the *endo-anti-exo* product as the minor isomer. While CsY zeolite enhances the selectivity to the *endo-anti-exo* isomer [29], the mesoporous material MCM-41 enhances the conversion to the *endo-anti-endo* isomer as has been shown in a previous work [30]. However, MCM-41 in the form of aluminosilicate that contains Brønsted sites enhances the retro-Diels–Alder reaction increasing the selectivity to the *endo-anti-exo* isomer. Therefore, the framework and extra framework composition of mesoporous materials and zeolite could be used to control the selectivity of the DAR of cyclopentadiene and *p*-benzoquinone.

In the present work, a series of large pore, pure silica zeolites (in which rate enhancement can only occur by spatial confinement) and the same structures but containing framework Brønsted or Lewis acid sites have been studied for the DAR between cyclopentadiene and *p*-benzoquinone. The effects of pore dimensions and catalyst composition on diffusivity and selectivity with respect to the retro-Diels–Alder reaction (retro-DAR) are discussed.

## Results and Discussion

**Scheme 1:**
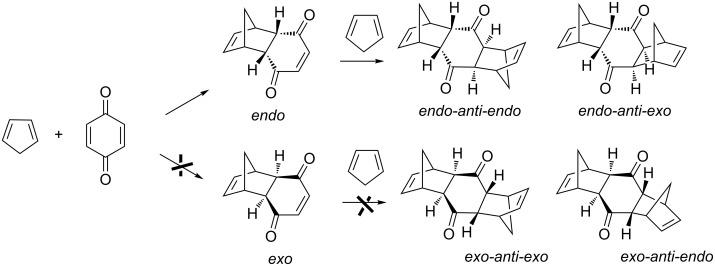
Distribution of products in the Diels–Alder reaction between cyclopentadiene and *p*-benzoquinone.

As it was described previously [30], the Diels–Alder reaction (DAR) between cyclopentadiene and *p*-benzoquinone follows the reactions outlined in Scheme 1.

As expected, the Diels–Alder cycloaddition provides the kinetically controlled *endo* isomer that very rapidly reacts with a second molecule of the diene to give again the kinetic *endo-endo* isomer. It is remarkable that neither the thermodynamic *exo* isomer nor the secondary *exo-endo* and *exo-exo* products were detected under our reaction conditions. Thus, the observed products, *endo-endo* and *endo-exo* are obtained in different ratio according to the reaction conditions. This ratio can change with the time since the retro-Diels–Alder reaction appears as a competitive reaction. In this way, the final molecular product can revert to the initial *endo* isomer, which in turn can react again with a new cyclopentadiene molecule. This is reflected in the distribution products by a decrease of the *endo-endo* isomer (kinetic control product) jointed to an increase of the *endo-exo* (thermodynamic control product).

### Influence of catalyst surface

**Figure 1:**
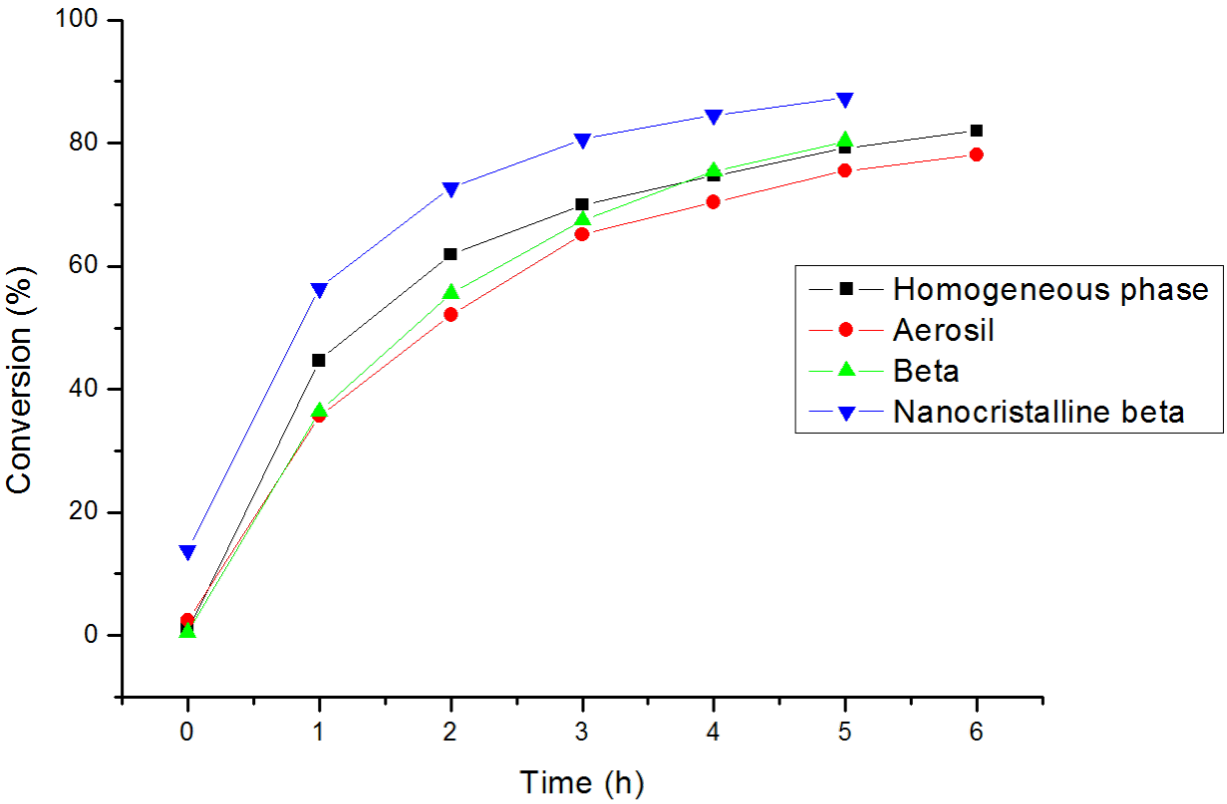
Conversion in the DAR catalysed by silica Beta zeolites and Aerosil.

We have seen that the DAR between cyclopentadiene and *p*-benzoquinone takes place thermally. The effect of confinement of the reactant within the pores of the catalyst can decrease the entropy of the activated complex [17–26,29–30] producing not only an increase of the reaction rate but also a modification of the selectivity. To study this effect, we have firstly carried out the reaction using a large pore Beta zeolite as catalyst. Thus, Figure 1 compares conversion results obtained for all silica Beta zeolite with that obtained during the thermal reaction or using Aerosil (amorphous non porous silica, BET surface area = 200 m^2^g^−1^) as potential catalyst. Practically no differences were found on reaction rate nor on product distribution when the reaction occurs on nonporous silica, with Beta zeolite or even in absence of any solid. Considering that Aerosil is an amorphous solid, these results indicate that the catalytic reaction with pure silica Beta zeolite, if any, should only occur on the catalyst surface and the porous structure has not any effect on the reaction. Another hypothesis to explain these results is that diffusion of the products through the channels, if ever formed inside, is strongly restricted and the products remain adsorbed within the pores. To evaluate this second hypothesis, ^13^C MAS NMR, elemental analysis and material balance were done, and the results obtained allow us to discard the accumulation of the reaction products inside the pores of the catalyst.

In order to check if the process is diffusion controlled within the pores of the zeolite and the reaction is mainly occurring on the external surface, the reaction was carried out with a pure silica nanocrystalline Beta (see Table 1). Table 1 shows differences between textural characteristics of all studied materials in this work. Figure 1 shows, an increase of the reaction rate when reducing the crystallite size of the zeolite, indicating that there is a reactant diffusion control within the pores of Beta zeolite and consequently the reaction is mainly occurring in an outer shell of the crystals. If this is so, and since the reaction rate increases with the pure silica nanocrystalline Beta zeolite with an external surface area not too different from Aerosil silica, we can conclude that a concentration effect within the pore mouth of the zeolite may be responsible for the reaction rate enhancement observed with pure silica nanocristalline beta.

**Table 1:** Textural characteristic of the studied materials.

Catalyst	Area (m^2^ g^−1^)^a^	Crystallite size (μm)^b^	Metal content (wt %)^c^	External surface (m^2^ g^−1^)^d^

Beta	457	0.5–1	–	24
Nanocrystalline Beta	595	0.015-0.02	–	100
Ti-Beta	454	1	1.2	25
Sn-Beta	470	1	1.6	30
Beta Si/Al = 13	518	0.1–0.2	2.8	34
Beta Si/Al = 50	484	0.2	0.9	50

^a^Area: Total area of the material per unit of mass. ^b^Crystallite size: Size of the crystalline material (aggregate of a large number of single crystals). It can vary from a few nanometers to several millimeters. ^c^Metal content: wt percentage of the metal content within the solid structure. ^d^External surface: External area of the material per unit of mass.

### Introduction of Lewis and Brønsted acid sites in the solids

We have prepared Ti-Beta [31], Sn-Beta [32] and Al-Beta (Si/Al = 50) [33] considering that Lewis acid catalyzes the DAR [1]. This effect is known to occur by complexation of the carbonyl group of the dienophile with the Lewis acid that increases the electron deficiency of the dienophile, reducing the energy gap.

**Figure 2:**
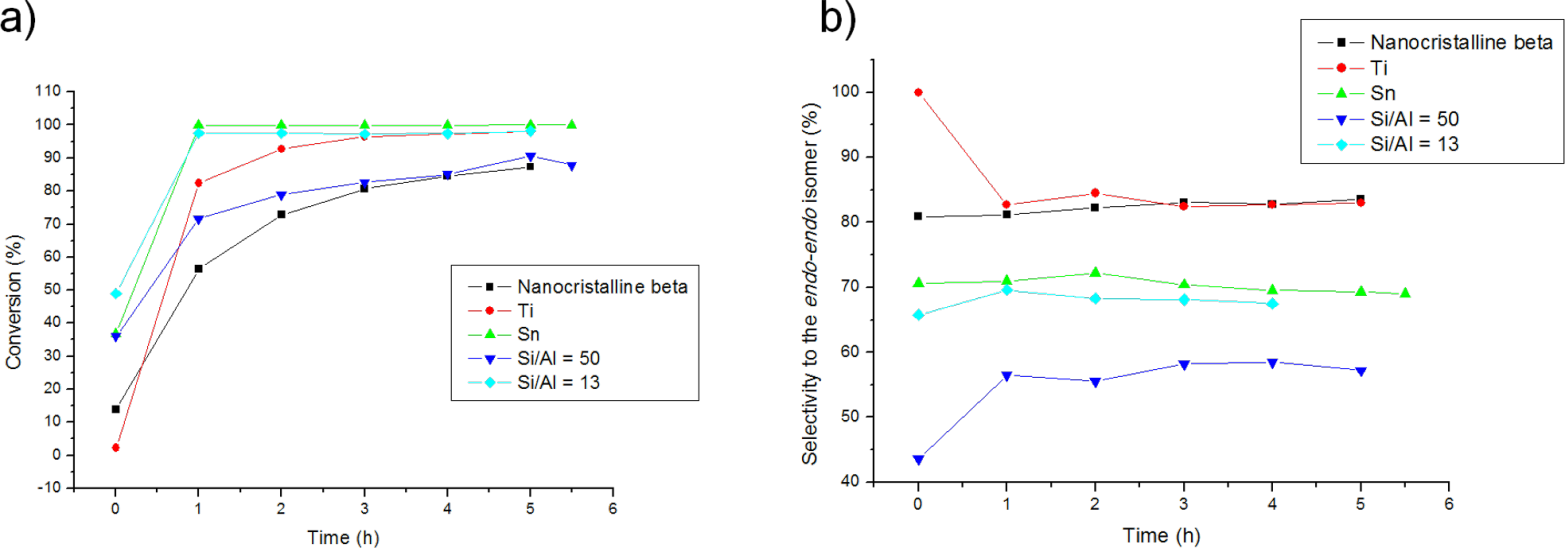
Effect of Lewis and Brønsted acid sites in the conversion (a) and selectivity (b) of the DAR.

The results presented in Figure 2a, b clearly show an important increase in activity due to the presence of Brønsted and, especially, of Lewis acid sites. Indeed, despite the fact that the crystallite size of Ti- and Sn-Beta zeolites is much larger than Al-Beta (Table 1), the former give higher conversions.

Importantly, the catalytic effect on the selectivity of the competing retro-Diels–Alder reaction, which produces an enhancement of the *endo-exo* isomer from the *endo-endo* (see Scheme 1), is much lower for Ti-Beta and even for Al-Beta zeolites than for MCM-41 [30] (see Figure 2a, b) that owing to the retro-DA reaction the selectivity of the *endo-endo* isomer decreases from 85% to 65% as we previously reported. [30]

**Figure 3:**
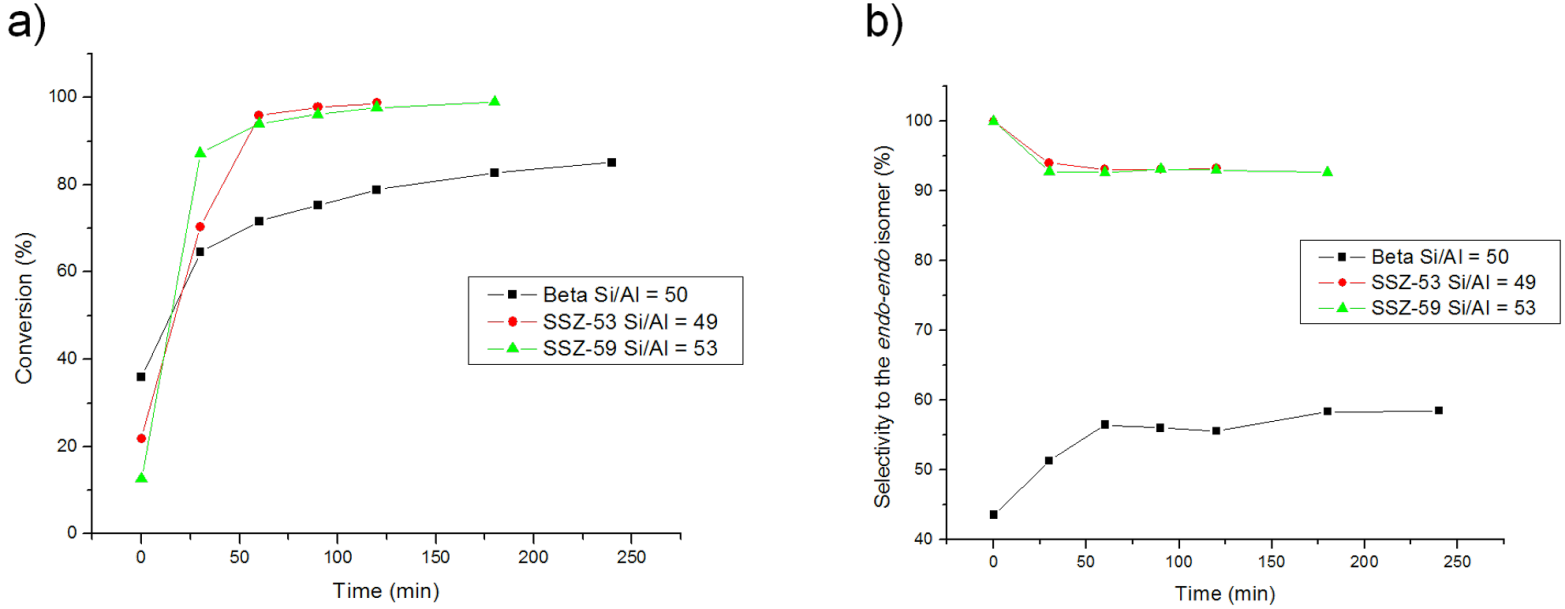
Effect of pore size in the conversion (a) and selectivity (b) of the DAR.

Considering the interesting application of beta zeolites as Lewis acid catalyst for Diels–Alder reactions in different fields, i.e., the formation of biofuels [34], it is important to get insight into the lack of catalytic activity of Beta for the retro-DAR, and elucidate whether this is a general effect with zeolites. Due to the diffusion limitations with Beta we have selected two extra-large pore zeolites, SSZ-53 (BET surface area = 377 m^2^/g) and SSZ-59 (BET surface area = 383 m^2^/g), with 1D pore system and a Si/Al ratio of 49 and 53, respectively. The results given in Figure 3 clearly show that the extra-large pore zeolites with pore diameters of 8.7 Å and 8.5 Å for SSZ-53 and SSZ-59, respectively, give higher conversions than Beta zeolite, despite the smaller crystallite size of the last. Interestingly, the retro-DAR was neither observed with the two zeolites with extra-large pores. Similarly to that produced with the silica nanocrystalline Beta zeolite a concentration effect within the extra-large pore mouth may be responsible of the reaction rate enhancement observed with SSZ-53 and SSZ-59.

Therefore, the results seem to confirm that the occurrence of retro-DAR as a competitive reaction not only depends on the presence of Brønsted centers as previously reported for materials with lower amounts of Al centers [28], but the structure of the material can play a determinant role. This represents an interesting observation since it will imply that, in principle, it should be possible to change the relative selectivity for DAR and retro-DAR working with micro or mesoporous catalysts.

**Figure 4:**
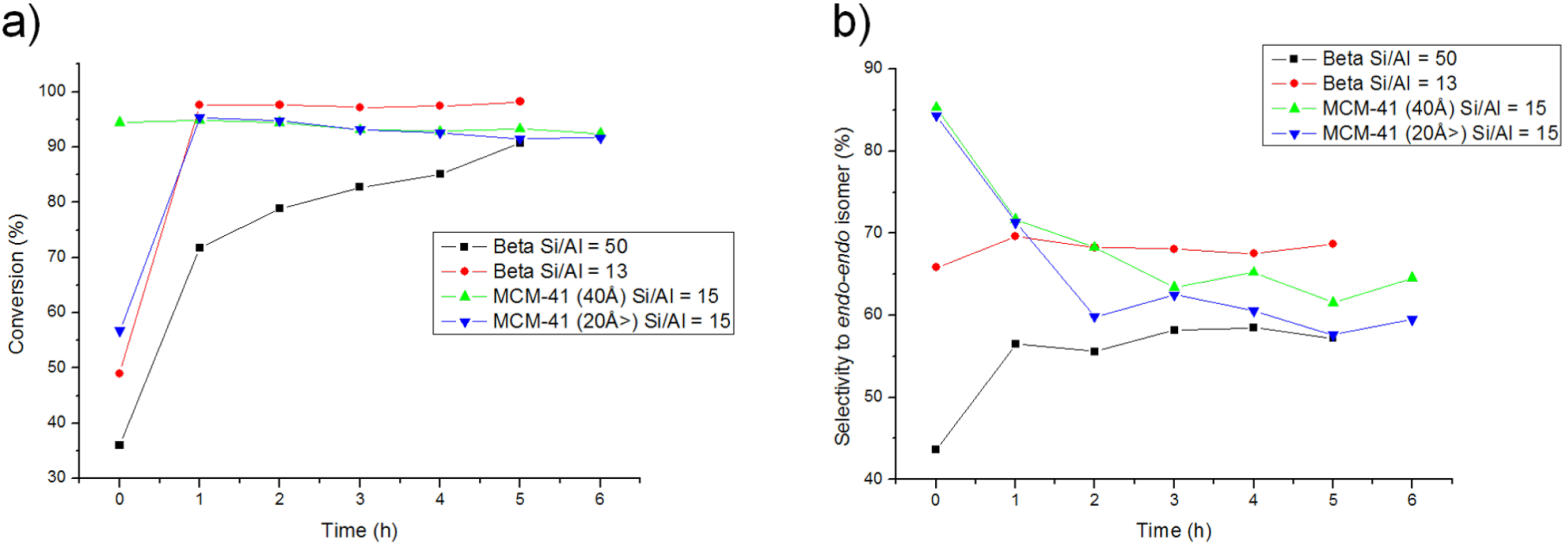
Comparison of conversion (a) and selectivity (b) of the DAR catalysed by Al-Beta zeolite and MCM-41.

Thus, Figure 4a,b compares conversion and selectivity to the *endo-endo* isomer with Al-Beta zeolite and the mesoporous MCM-41 material previously studied [30] both with very close Si/Al ratios. It can be observed that both samples give the same conversion, but different selectivity behavior.

In the case of the microporous catalyst, Al-Beta zeolite, the selectivity to the *endo-endo* isomer remains constant with time, while with MCM-41 that is formed by larger channels, a continuous decrease of the *endo-endo* with time occurs and the thermodynamically controlled *endo-exo* product increases. The retro-Diels–Alder is a consecutive reaction that produces the thermodynamic product and it would occur if there is a certain confinement within the pores.

Thus, it was thought that if retro-DAR occurs in MCM-41 (40 Å), if the pore size is decreased, then this reaction should be enhanced because of a certain confinement effect through the reactants. As it can be observed in Figure 4a,b, when the reaction was carried out with a mesoporous material of ≈20 Å instead of 40 Å but with a similar Si/Al ratio, the retro-DAR was enhanced, illustrating a certain confinement effect within the pores.

**Figure 5:**
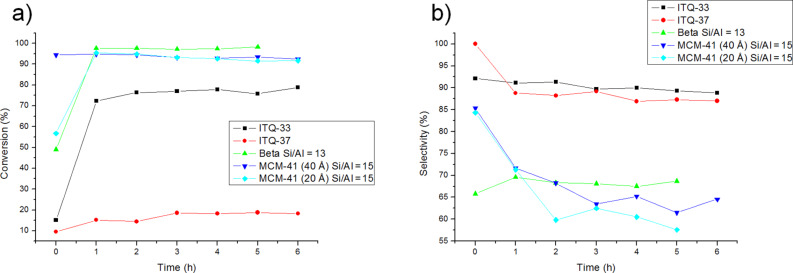
Comparison of conversion (a) and selectivity (b) of the DAR catalysed extra-large pore 3D zeolites.

Two extra-large pore 3D zeolites with pore diameters of 1.2 (ITQ-33) [35] and 1.9 nm (ITQ-37) [36] have also been tested. These are aluminosilicogermanates that, as the previously tested Al-zeolites or the Al-MCM-41 material, present Brønsted acidity. Interestingly, the pore diameters of ITQ-33, and more so ITQ-37 are close to the pore of the mesoporous MCM-41 presented above with 2.0 nm. There is then a unique occasion to compare the catalytic behavior of an amorphous and a crystalline molecular sieve with practically the same pore diameter (Figure 5a,b).

As observed in Figure 5a,b, the crystalline structure of zeolites ITQ-33 and ITQ-37 do not favor neither the Diels–Alder cycloaddition between cyclopentadiene and *p*-benzoquinone, nor the retro-Diels–Alder reaction. This result suggests that the reaction takes place on the surface of the material and the pore structure does not have any influence on the reaction rate, neither for the DAR nor for the retro-DAR.

**Figure 6:**
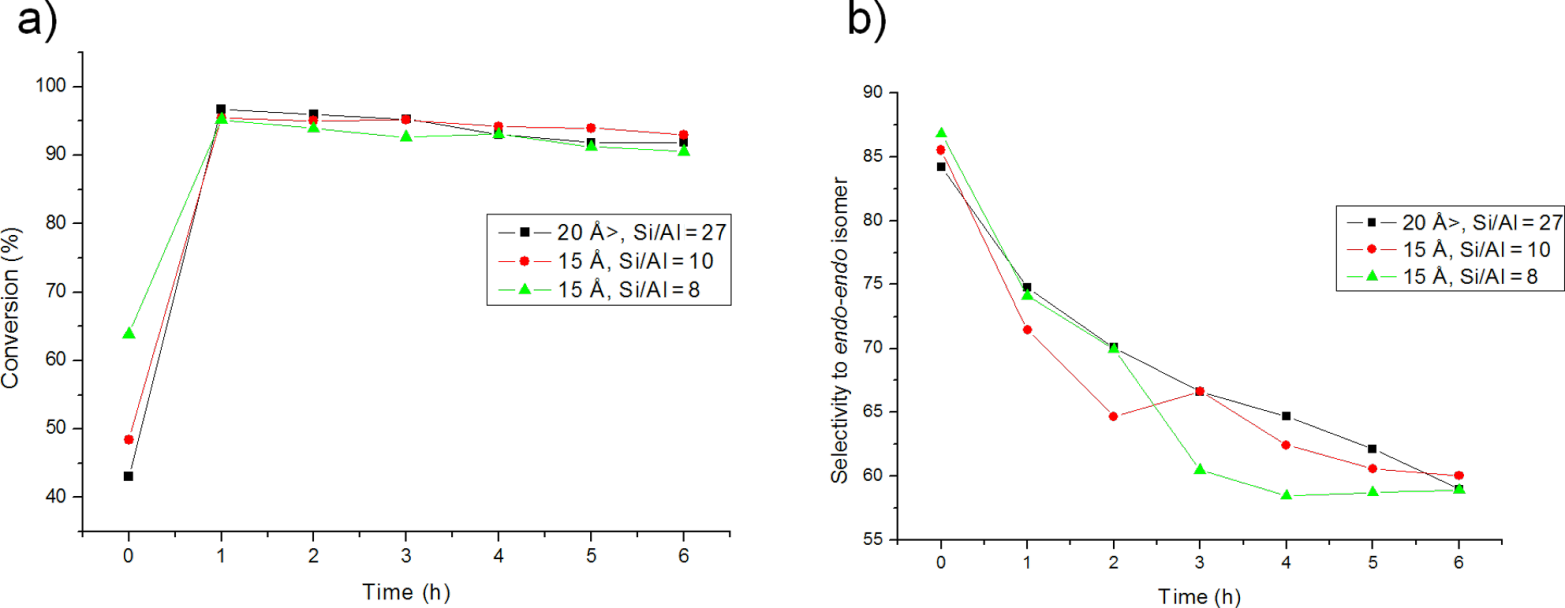
Effect of the Si/Al ratio in the conversion (a) and selectivity (b) of the DAR.

To further prove the effect of the structure, the reaction was carried out in presence of MCM-41 materials with different Si/Al ratios, and similar pore diameter. The results are collected in Figure 6a,b. As it could be expected no differences were found in the conversion. Meanwhile, in the case of the selectivity it is possible to observe that increasing the Al content and lowering the pore size produces an increase in the selectivity of the *endo-exo* isomer. However, this effect is much more marked when the pore size decreases.

**Figure 7:**
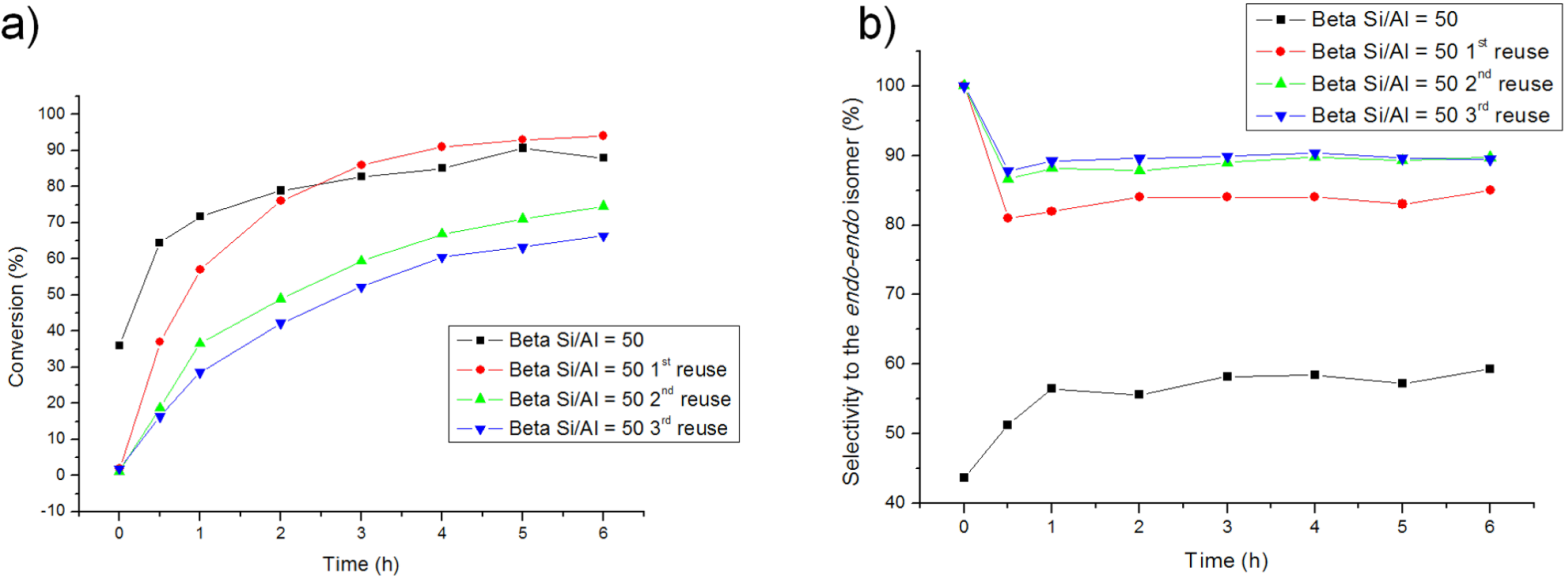
Effect of the reutilization of the catalysts in the conversion (a) and selectivity (b) of the DAR.

Finally, to conclude the catalytic study of the reaction between cyclopentadiene and *p*-benzoquinone in presence of Beta zeolites, the ability of reuse of Beta Si/Al = 50 was examined. As shown in Figure 7a,b the activity of the catalyst decreases in some extension after repeated recycling. As expected for a less active catalyst, conversion falls partly while the selectivity to the kinetically controlled *endo-endo* isomer rises after recycling.

## Conclusion

In this work the DAR between cyclopentadiene and *p*-benzoquinone has been proved to take place on the catalyst surface when the reaction is carried out in presence of microporous materials, obtaining better results when a smaller crystal size catalyst is used.

When Lewis and Brønsted sites are inserted in the material structure, an improvement of the conversion degree is obtained as it occurs when MCM-41 and ITQ-2 were used [30]. However, a clear change in the selectivity behavior is observed. None of the used metals showed a retro-DAR enhancing reactivity, even Al, the best hydrogen-bond-donating agent. This result implies that the competitive retro-DAR takes place not only due to the capability to act as Brønsted sites of metallic centers, but also due to the structure of MCM-41 and ITQ-2. This effect can be used in order to obtain a selective product or the other isomer as a result of the chosen catalyst: The more Brønsted sites and the more confinement of the reactants, the more retro-DAR will be observed.

## Experimental

### Catalyst preparation

Beta zeolites [pure silica Beta, Beta (Ti), Beta (Sn) and Beta (Al)] were prepared according to [31–33], using tetraethylammonium hydroxide as template, tetraethyl orthosilicate (TEOS) as silica source and Ti(IV) ethoxide, SnCl_4_·5H_2_O and metal Al as sources of heteroatoms. SSZ-53 and SSZ-59 were synthesized according to the procedures described in the literature [37–41]. The textural characteristics of the catalysts are given in Table 1.

### Catalytic tests

In a similar manner as described in [30], after being activated at 250 °C under vacuum (10^−2^ mm Hg), 250 mg of the corresponding calcined material were introduced into a two necked bottom flask under N_2_. Then, 108 mg of *p*-benzoquinone (1.0 mmol) and 10.0 mL of CDCl_3_ were added. The mixture was stirred at room temperature for a few minutes and 0.2 mL of freshly distilled cyclopentadiene (3.0 mmol) were added with a syringe, being this moment considered *t* = 0 h. The system was heated at 60 °C and samples were taken every hour, being directly analyzed by ^1^H NMR.

Reaction products were isolated by HPLC using mixtures of H_2_O/MeOH/MeCN (45:50:5). Identification of these products was carried out by NMR techniques (^1^H, ^13^C, DEPT, COSY, HETCOR and NOE) being the spectral data fully coincident with those reported in the literature [42].

Conversion values for *endo-endo* and *endo-exo* products are always referred to conversion from the *endo* adduct. All compounds were previously described and fully characterized [30].
